# Vaginal dysbiosis seems associated with hrHPV infection in women attending the Dutch Cervical Cancer Screening Program

**DOI:** 10.3389/fcimb.2024.1330844

**Published:** 2024-03-13

**Authors:** Anne J. M. Loonen, Femke Verhagen, Ilse Luijten-de Vrije, Marjolein Lentjes-Beer, Cornelis J. Huijsmans, Adriaan J. C. van den Brule

**Affiliations:** ^1^ Lab for Molecular Diagnostics, Pathologie-DNA, Jeroen Bosch Hospital, ‘s-Hertogenbosch, Netherlands; ^2^ Research Group Applied Natural Sciences, Fontys University of Applied Sciences, Eindhoven, Netherlands

**Keywords:** high risk HPV, cervical dysplasia, vaginal dysbiosis, Cervical Cancer Screening Program, vaginome

## Abstract

Human papillomavirus (HPV) is a sexually transmitted virus, which infects approximately 80% of all men and women at some time in their lives. Usually, the infection is resolved successfully by the body’s immune system. Persistent infection with high-risk HPV (hrHPV) is necessary but not sufficient for cervical cancer development, and additional factors, such as the vaginal microbiome (vaginome), are thought to be involved. The aim of this study is to investigate whether either vaginal dysbiosis (imbalance in vaginal bacterial composition) or sexually transmitted pathogens, e.g., *Chlamydia trachomatis* (CT), are possible cofactors for hrHPV infection and HPV-induced cervical dysplasia in asymptomatic women attending the Dutch Cervical Cancer Screening Program. In this study, 492 hrHPV-positive and 500 hrHPV-negative cervical smears from women attending the Screening Program were included. Age and cytology were known for the hrHPV-positive samples. All cervical smears were diluted in Aptima^®^ specimen transfer medium and tested with Aptima^®^ transcription-mediated amplification assays targeting CT*, Neisseria gonorrhoeae* (NG)*, Mycoplasma genitalium* (MG)*, Candida* spp. (CS), *C. glabrata* (CG)*, Trichomonas vaginalis* (TV), and bacterial vaginosis (BV). The prevalences of CT, NG, MG, CS, CG, TV, and BV in this cohort were found to be 1.9%, 0.0%, 1.7%, 5.4%, 1.4%, 0.1%, and 27.2%, respectively. When comparing HPV groups, it was found that CT, MG, and BV had a significantly higher prevalence in hrHPV-positive smears as compared with hrHPV-negative samples (for all *p* < 0.001). No significant differences were found when comparing different age groups and cytology outcomes. In conclusion, vaginal dysbiosis seems associated with hrHPV infection in women attending the Dutch Cervical Cancer Screening Program.

## Introduction

Cervical cancer is the fourth most common cancer among women, with an estimated 604,000 new cases and 342,000 deaths around the world in 2020. Approximately 90% of the new cases and deaths in 2020 occurred in low- and middle-income countries (Sung et al., 2021). Persistent infection by certain oncogenic high-risk (hr) human papillomavirus (HPV) types is firmly established as the necessary cause of most premalignant and malignant epithelial lesions of the cervix (Bosch et al., 2002). However, few HPV infections progress to cervical cancer and most HPV infections are cleared by the host immune system (Castellsague, 2008). The reason(s) why hrHPV infection progresses to high-grade premalignant lesions and cancer in some women but not in others is unknown. However, the vaginal microbiome (vaginome) is suggested to play a role (Castanheira et al., 2021; Dareng et al., 2016; Fethers et al., 2008; Gao et al., 2013; Kyrgiou et al., 2017; Laniewski et al., 2020; Lee et al., 2013; Musa et al., 2023). The normal vaginome in the female reproductive tract typically reveals *Lactobacillus* species as the predominance genus, which helps to promote a healthy vaginal environment (Gillet et al., 2011). The vaginome can currently be sorted into five community state types (CSTs) based on microbial community composition and abundance. CSTs I, II, III, and V are dominated by *L. crispatus*, *L. gasseri*, *L. iners*, and *L. jensenii*, respectively. The main mechanisms with which *Lactobacillus* spp. protect the female reproductive system include (i) competition with pathogens for the vaginal epithelium, (ii) progression of epithelial integrity by upregulating tight-junction proteins, (iii) prevention of growth of pathogens by lactic acid production, (iv) production of bacteriocins and hydrogen peroxide which have an antimicrobial effect, (v) development of the autophagy of cells infected by pathogens and help their elimination, and (vi) modulation of local defense (Sharifian et al., 2023).

CST IV is dominated by mixed anaerobes similar to those found in bacterial vaginosis (BV) (reflects a change in the normal balance of vaginal bacteria, not an infection) and predisposes women to infection by HPV (Gao et al., 2013). Also, presence of pathogenic microorganisms such as *Chlamydia trachomatis* (CT)*, Neisseria gonorrhoeae* (NG), *Mycoplasma genitalium* (MG), and *Trichomonas vaginalis* (TV) in the vagina seem to be associated with the persistence of oncogenic hrHPV infection (Lazenby et al., 2014; Silins et al., 2005; Ye et al., 2018).

The Dutch Cervical Cancer Screening Program focuses on women between the ages of 30 and 60 years. The cervical smear (brush) is placed in PreservCyt medium and subsequently tested for the presence of hrHPV by a nucleic acid amplification test (NAAT). If hrHPV is detected, cytology analysis using the Papanicolaou (Pap) staining is used to screen for precancerous cells (www.rivm.nl/en/cervical-cancer-screening-programme).

A commonly used commercially available NAAT for hrHPV and other sexually transmitted infections (STIs) are Aptima^®^ assays, which can be used on the fully automated Hologic Panther system. Aptima^®^ assays are based on (real-time) transcription-mediated amplification (TMA) (Carter et al., 2020).

NAAT for detection of STIs in women is mostly effective when using vaginal swabs (Coorevits et al., 2018). Cervical smears are used to screen women for hrHPV, and few studies have shown the possibility of detecting sexually transmitted infections (STIs) such as CT and NG from this sample type (Hawthorne et al., 2005; Koumans et al., 2003).

In this study, we analyzed the prevalence of BV and STIs, such as, CT, NG, MG, *Candida* spp. (CS), *C. glabrata* (CG), and TV in women attending the Dutch Cervical Cancer Screening Program. Furthermore, we explored the relationship between the presence of these vaginome abnormalities and hrHPV infection.

## Materials and methods

### Ethics statement

This study was approved by the Medical Ethics Committee Brabant (NW2022-04), the Internal Medical Ethical Committee at the Jeroen Bosch Hospital (2021.11.10.01), and the Dutch Institute for Public Health and Environment (RIVM). After routine hrHPV screening, cervical smears were anonymized, coded, and stored in the Jeroen Bosch Hospital biobank for future use. Informed consent was checked via an opt-out procedure (remaining clinical material is allowed to be used for scientific research unless the client actively refuses the reuse of their sample).

### Analytical samples

*Lactobacillus crispatus* was cultured in 5 mL of MRS broth in an Anaerocult^®^ system (VWR) at 37°C for 48 h. Subsequently, fresh MRS broth was inoculated with 10% volume of cultured *L. crispatus* and incubated for 20 h at 37°C and 5% CO_2_ atmosphere to the exponential growth phase (log phase), measured with a spectrophotometer (600 nm). SiHa cells were cultured in MEM50 medium (Thermo Fisher Scientific) with 10% fetal bovine serum (FBS, Biowest) and 1% penicillin–streptomycin (Biowest). A range of 0–10^5^ colony-forming units (CFU)/mL log phase *L. crispatus* were diluted in Aptima tubes with Specimen Transfer Medium (STM) (Hologic B.V.) and vials with PreservCyt medium (20 mL, Hologic B.V.), with and without 10^4^ SiHa cells. SiHa cells were added to create a spike sample resembling a cervical smear. Subsequently, 1 mL from spiked PreservCyt medium was transferred to Aptima^®^ specimen transfer tubes, after which all Aptima^®^ specimen transfer tubes were tested with the Aptima^®^ BV assay on the Panther (Hologic B.V.) according to manufacturers’ instructions.

### Clinical samples

**Figure 1:**
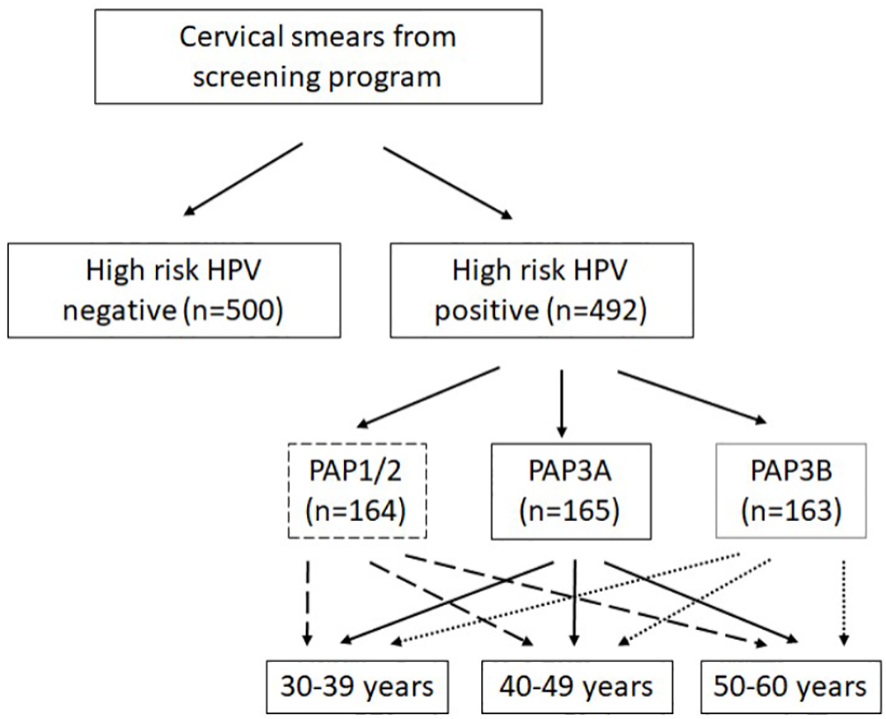
Overview of the study cohort. From hrHPV-positive sample cytology score (Papanicolaou, PAP); the age of the client was registered. From hrHPV-negative cervical smears, only HPV status was documented. High-risk HPV presence was tested in the Cervical Cancer Screening Program (Roche Cobas).

At the time cervical smears were collected (2020–2021), the Jeroen Bosch Hospital was one of five HPV-screening laboratories in The Netherlands, responsible for the South East region of The Netherlands. Cervical smears were collected and stored in PreservCyt medium at room temperature in the biobank. Cervical smears were tested for hrHPV presence with the Cobas^®^ HPV assay (Roche). When hrHPV-positive results were obtained, those cervical smears were also used for cytology by using PAP staining. If a sample tested negative for hrHPV, no cytology was performed. The Pap smear can result in six different outcomes: Pap0 (inconclusive results), Pap1 (normal cytology), Pap2 (Atypical Squamous Cells of Undetermined Significance (ASC-US)), Pap3a or 3b (low (3a) to moderate high (3b) grade squamous intraepithelial lesion (LSIL/HSIL)), Pap4 (HSIL), and Pap5 (cervical cancer). Cytology samples were scored from PAP1 to a maximum score of PAP3B. From hrHPV-positive samples, the cytology score and age of the client were registered in the biobank database. From hrHPV-negative cervical smears, only HPV status was documented (the age of women in this group is unknown). HPV vaccination status is not known of both hrHPV-positive and -negative women. The samples used in this study are shown in **Figure 1**: 500 HPV-negative samples are randomly collected; 492 HPV-positive smears were grouped on age and contained an equal amount of samples from women aged 30–39 years, 40–49 years, and 50–60 years. Furthermore, different categories of cytology diagnosis were included (according to either PAP1 and 2, PAP3A, or PAP3B). See **Figure 1** for a detailed overview.

### Aptima^®^ assays

The TomCat (Hologic B.V.) was used to automatically transfer 1 mL from each cervical smear (ThinPrep vials with PreservCyt medium) to Aptima^®^ specimen transfer tubes (Hologic B.V.). Aptima^®^ assays are based on the isothermal amplification of RNA by reverse transcription and subsequent generation of numerous transcripts by RNA polymerase. These RNA copies are hybridized with a complementary oligonucleotide probe for detection *via* a fluorescent-labeled molecular beacon. We made use of the bacterial vaginosis (BV), *Chlamydia trachomatis* (CT),/*Neisseria gonorrhoeae* (NG), *Mycoplasma genitalium* (MG), Candida spp., and *Trichomonas vaginalis* (vulvovaginal candidiasis and trichomoniasis CV/TV) assays on the Hologic Panther. The BV assay detects *L. gasseri*, *L. crispatus*, and *L. jensenii* as a group (one signal) together with *Gardnerella vaginalis* and *Atopobium vaginae*. The assays were performed conform the manufacturer’s instructions.

### Data analysis

Results were analyzed with SPSS (IBM, v28). To assess differences in the trend of the proportions of BV or STIs in the hrHPV-positive and -negative groups, the chi-square test was used. When the results did not fit the conditions of the chi-square test, the Fisher’s exact test was used. A *p* value of ≤0.05 was considered statistical significant.

## Results

### Quality check of cervical smears and *in vitro* sensitivity of the bacterial vaginosis assay

Some pilot tests were necessary to evaluate the technical feasibility of conducting a study to detect BV and several STIs from long-term stored cervical smears in PreservCyt medium with Aptima^®^ assays. One major problem with nucleic acid amplification tests such as the Aptima^®^ TMA technology is the presence of potential inhibitors in the biological samples, which can lead to false-negative results.

First, a confirmation panel was selected which consisted of 50 hrHPV-positive (previously tested with Cobas HPV assay (Roche)) and 5 hrHPV-negative cervical smears to perform a quality check on the stored biobank samples. 1 mL was transferred to Aptima^®^ tubes with STM and subsequently tested with the Aptima^®^ HPV assay. Results are shown in **Table 1**. Of the 55 samples tested, 48 were confirmed (87.3%). In total, seven hrHPV-positive cervical smears tested as hrHPV negative with the Aptima HPV assay, six with a PAP1 status, and one with a PAP3A status. These results were in agreement with our previous published study (Huijsmans et al. [DuSC) (Huijsmans et al., 2016)] in which the concordance between different HPV detection methods was higher in cervical dysplasia than in normal cytology samples, due to higher HPV viral load in the first group of samples.

**Table 1:** Results of the confirmation panel with the Aptima^®^ HPV assay.

Sample	Aptima hrHPV positive	Aptima hrHPV negative
hrHPV-positive PAP1 (n = 20)	14 (70%)	6 (30%)
hrHPV-positive PAP2 (n = 10)	10 (100%)	0 (0%)
hrHPV-positive PAP3A (n = 10)	9 (90%)	1 (10%)
hrHPV-positive PAP3B (n = 10)	10 (100%)	0 (0%)
hrHPV-negative (n = 5)	0 (0%)	5 (100%)

Samples were previously tested by the Cobas^®^ HPV assay. The panel consisted of n = 20 hrHPV-positive PAP1, n = 10 hrHPV-positive PAP2, n = 10 hrHPV-positive PAP3A, n = 10 hrHPV-positive PAP3B, and n = 5 hrHPV-negative cervical smears.

**Figure 2:**
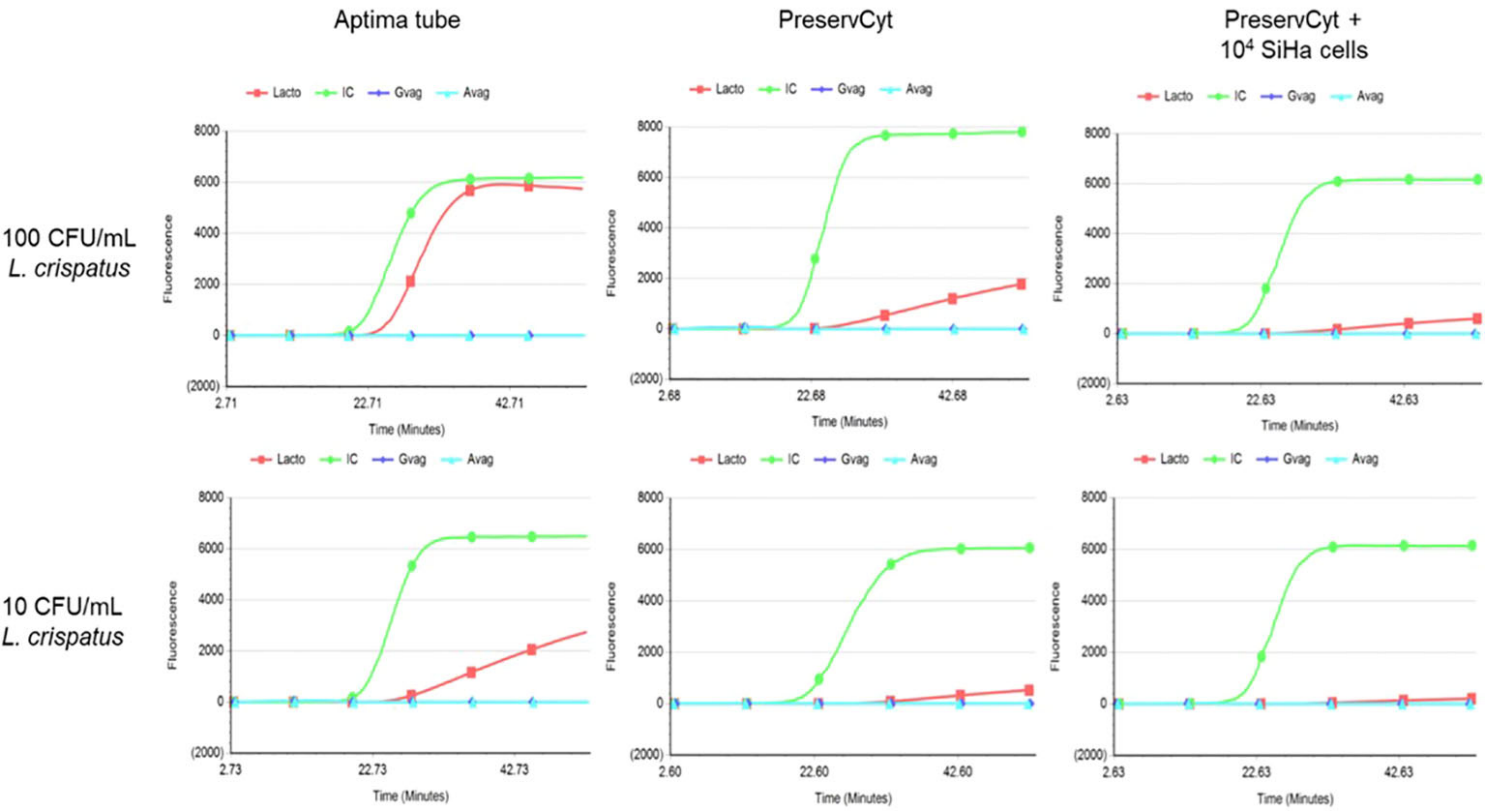
Results of *Lactobacillus crispatus*-spiked Aptima^®^ Specimen Transfer Medium (left), PreservCyt without SiHa cells (middle), and PreservCyt with 10^4^ SiHa cells (right). The upper graphs represent 100 CFU/mL, and the lower graphs represent 10 CFU/mL. On the x-axis time in minutes and on the y-axis fluorescent signal are depicted. CFU; colony-forming units, IC (green); internal control, Lacto (red) *Lactobacillus* spp., Gvag (dark blue); *Gardnerella vaginalis*, Avag (light blue); *Atopobium vaginae*.

Second, to evaluate the analytical sensitivity of one of the bacteria detected by the BV assay, *Lactobacillus crispatus* was spiked in a range of 0 to 10^5^ CFU/mL in Aptima^®^ STM medium and PreservCyt medium (20 mL) with and without 10^4^ SiHa cells (cervical cells, HPV16 positive). 1 mL of this spiked PreservCyt was transferred to Aptima^®^ STM for measurement on the Panther platform. See **Figure 2** for the results. Both 100 and 10 CFU/mL *L. crispatus* can be detected from directly spiked Aptima^®^ STM, with approximately 6,000 and 2,000 RLU, respectively. The maximum RLU value of 100 CFU/mL spiked *L. crispatus* in PreservCyt medium was 2,000. SiHa cells’ presence in the PreservCyt sample with 100 CFU/mL *L. crispatus* resulted in a signal of approximately 800 RLU.

These results demonstrate that PreservCyt medium seems a suitable medium for detection of *L. crispatus* from cervical smears as a limit of detection of 143 CFU/mL is described in the manual of the Aptima^®^ BV assay. However, loss in sensitivity due to an additional dilution step (20×) must be taken into account.

### Screening of cervical smears

The Aptima^®^ BV, CT/NG, MG, and CV/TV assays were used to screen n = 992 cervical smears from the Dutch Cervical Cancer Screening Program, n = 500 were hrHPV negative, and n = 492 were hrHPV positive (Cobas^®^ HPV assay, Roche). In total, n = 270 samples were positive for BV resulting in a prevalence of 27.2% in this cohort. The prevalence of BV in the hrHPV-positive group was significantly higher (*p* < 0.001) as compared with the prevalence in the hrHPV-negative group, 36.6% versus 18.0%, respectively (see **Table 2** for results). Clinical findings regarding the presence of clue cells or increased pH were not known and could not be compared with BV-positive results. There were 19 samples that tested positive for CT (prevalence of 1.9%), and none were positive for NG. In total, 17/19 positive CT samples were detected in hrHPV-positive smears resulting in a significant difference (*p* < 0.001) of CT prevalence between hrHPV-positive and -negative groups. For MG, 17 positive results were found in this cohort (prevalence of 1.7%) of which 15 clustered with hrHPV positivity. Also for the prevalence of MG, a significant difference (*p* < 0.001) was observed between the hrHPV-positive and -negative groups. *Candida* spp. were detected with a prevalence of 5.4%, *C. glabrata* was detected in 1.4% of cervical smears, and one positive result was found for TV. No significant differences were found between hrHPV-positive and -negative groups for these microorganisms. Subsequently, for the hrHPV-positive group, different age groups and cytology outcomes were compared; however, no significant differences were found. For the different age and PAP groups, *p* values were found in the range of *p* = 0.092 to *p* = 0.900. For NG and TV, no statistical analysis could be performed as 0 and 1 positive sample was found, respectively.

**Table 2:** Prevalence and relevance of different urogenital pathogens in relation to hrHPV status.

	Cohort	hrHPV positive	hrHPV negative	Chi-square test
Bacterial vaginosis	27.2% (270/992)	36.6% (180/492)	18.0% (90/500)	*p* < 0.001
*Chlamydia trachomatis*	1.9% (19/992)	3.4% (17/492)	0.4% (2/500)	*p* < 0.001
*Neisseria gonorrhoeae*	0.0% (0/992)	0.0% (0/492)	0.0% (0/500)	ND
*Mycoplasma genitalium*	1.7% (17/992)	3.0% (15/492)	0.4% (2/500)	*p* < 0.001
*Candida* spp.	5.4% (53/990)	5.9% (29/490)	4.8% (24/500)	*p* = 0.434
*Candida glabrata*	1.,4% (14/990)	1.0% (5/490)	1.8% (9/500)	*p* = 0.299
*Trichomonas vaginalis*	0.1% (1/990)	0.1% (1/490)	0.0% (0/500)	*p* = 0.312

Chi-square test was performed to analyze if there were significant differences between groups.

ND, not determined; *Candida* spp., *C. albicans, C. tropicalis, C. parapsilosis, C. dubliniensis*.

## Discussion

The lifetime risk of acquiring any HPV infection exceeds 80%; however, the lifetime risk of developing invasive cervical cancer is much lower than 1% (Kyrgiou et al., 2017). The vaginome seems to play an important role in the persistence and clearance of HPV in the human vagina (Castanheira et al., 2021; Sharifian et al., 2023). In this study, we explored the association between the presence of BV and different STIs in relation to hrHPV infection, cervical cytology (PAP scores) and age in women who participated in the Dutch Cervical Cancer Screening Program.

The results of this study showed a statistically significant difference (*p <* 0.001) for the prevalence of BV, CT, and MG in hrHPV-positive cervical smears compared with HPV-negative samples. No association was found between different cytology categories and age groups in this cohort of women. Multiple studies have shown the association between vaginal dysbiosis (e.g., BV) and vaginal infections with hrHPV, vulvovaginal candidiasis (VVC), and STIs, e.g., CT/NG and MG infection (Sharifian et al., 2023; Chee et al., 2020). BV is characterized by a change in the vaginome dominated by *Lactobacillus* species to a polymicrobial anaerobe-dominated vaginome that includes *Gardnerella vaginalis, Atopobium vaginae*, and other bacteria (Onderdonk et al., 2016). The Aptima BV assay targets *Lactobacillus* species as a group (*L. gasseri, L. crispatus*, and *L. jensenii*), *Gardnerella vaginalis*, and *Atopobium vaginae* and uses an algorithm to report a qualitative result for BV based on detection of target organisms [Hologic B.V.]. In healthy women, CST I and V are dominant microbiota. BV is often referred to CST IV and linked to increased risk for STIs such as hrHPV infection (Sharifian et al., 2023). Women with BV are more susceptible to HPV due to the increased pH value in the vagina and missing *Lactobacillus* species (Fu and Zhang, 2023). Furthermore, disruption of vaginome homeostasis can threaten health conditions due to increasing host susceptibility to infections (Aviles-Jimenez et al., 2017).

In addition, we analyzed the prevalence of BV and sexually transmitted infections (STIs) in this cohort of women who participated in the Dutch Cervical Cancer Screening Program. In this cohort, a prevalence of 27.2%, 1.9% 1.7%, 5.4%, and 1.4% was found for BV, CT, MG, *Candida* spp., and *C. glabrata*, respectively. The prevalence of BV is reported to be between 10% and 50% worldwide (Amaya-Guio et al., 2016; Borgdorff et al., 2017; Vahidnia et al., 2015). This range can be contributed to a wide variety between study cohorts, which differ based on age groups, ethnicity, and presence/absence of symptoms. Redmond et al. performed a systematic review to estimate the Chlamydia prevalence in Europe and non-European high-income countries. They concluded that Chlamydia prevalence ranged from 0.6% to 10.7% in women. In the Netherlands, a prevalence for CT of approximately 3.5% was found (Redmond et al., 2015). In contrast, there is limited data on the prevalence of MG and TV in The Netherlands and the available data come from mixed populations. In a study by Yusuf et al., in which samples were used from different care settings (general practitioners, STI clinics, and hospitals), the prevalence of MG was described to vary between 1.4% and 6.7% and for TV between 0.6% and 4.5% (Yusuf et al., 2023). In the Netherlands, the most reliable data for the incidence of *Candida* spp. comes from general practitioners. Those numbers indicate a prevalence between 1.5% and 4.2% per year (www.soaaids.nl). We have shown in the spike experiments with *L. crispatus* that sensitivity decreases in Aptima^®^ assays by using cervical smears (take dilution factor into account). However, our findings in regard to prevalence of the tested microorganisms are in line with literature.

Cervical smears were used from our biobank. An initial evaluation of hrHPV positivity on a subset of these samples with the Aptima HPV assay showed a concordance of 87.3% with the Cobas HPV assay (Roche), indicating that the quality of the samples stored in the biobank was still acceptable and conform results published by Huijsmans et al. who showed that when comparing different HPV assays, hrHPV-positive samples with normal cytology (PAP1) show interassay agreement of approximately 50% (Huijsmans et al., 2016), most likely due to lower HPV viral load in these samples. However, for hrHPV-positive samples with abnormal cytology (PAP3B and higher), concordance is between 90% and 100%.

A possible limitation of this study is that the cohort used is not a reflection of the Dutch screening population. In The Netherlands, only 8%–10% hrHPV-positive samples are found (Huijsmans et al., 2016; de Kok and Aitken, 2022). In this cohort, 50% of the samples are hrHPV positive and a substantial amount of samples is also categorized as PAP3B. Most likely, the HPV-negative women are of random age but might not perfectly match the groups in the hrHPV-positive group. Furthermore, only one unique sample per client was analyzed. It would be interesting to look into follow-up samples of women attending the Screening Program to investigate with, e.g., next-generation sequencing, which specific (set of) microorganism(s) are associated with hrHPV infection, and development of cervical dysplasia or even cervical cancer.

In conclusion, vaginal dysbiosis seems associated with hrHPV infection in women attending the Dutch Cervical Cancer Screening Program. Especially, BV, CT, and MG are risk factors for hrHPV infection. We have shown that one cervical smear (in PreservCyt medium) may enable gynecologists to simultaneously monitor for both hrHPV, urogenital infections, and cervical lesions (cytology) within a single specimen. Future additional analyses of the vaginome by using next-generation sequencing will increase our knowledge of their role in health and disease and could have important diagnostic and therapeutic implications.

## Data availability statement

The raw data supporting the conclusions of this article will be made available by the authors, without undue reservation.

## Ethics statement

The studies involving humans were approved by Medical Ethical Committee Brabant. The studies were conducted in accordance with the local legislation and institutional requirements. The human samples used in this study were acquired from a by-product of routine care or industry. Written informed consent for participation was not required from the participants or the participants’ legal guardians/next of kin in accordance with the national legislation and institutional requirements.

## Author contributions

AL: Formal Analysis, Methodology, Project administration, Supervision, Validation, Writing – original draft, Writing – review & editing. FV: Investigation, Writing – review & editing. IL-d: Investigation, Writing – review & editing. ML-B: Methodology, Validation, Writing – review & editing. CH: Methodology, Writing – review & editing. AB: Formal Analysis, Methodology, Resources, Supervision, Validation, Writing – review & editing.
